# Molecular Dynamics Simulation on Behaviors of Water Nanodroplets Impinging on Moving Surfaces

**DOI:** 10.3390/nano12020247

**Published:** 2022-01-13

**Authors:** Hao Zhang, Ling Pan, Xuqing Xie

**Affiliations:** School of Mechanical Engineering and Automation, Fuzhou University, Fuzhou 350108, China; hao_capable@126.com (H.Z.); n190227107@fzu.edu.cn (X.X.)

**Keywords:** water nanodroplets, molecular dynamics, dynamical behaviors, translation surface, vibration surface

## Abstract

Droplets impinging on solid surfaces is a common phenomenon. However, the motion of surfaces remarkably influences the dynamical behaviors of droplets, and related research is scarce. Dynamical behaviors of water nanodroplets impinging on translation and vibrating solid copper surfaces were investigated via molecular dynamics (MD) simulation. The dynamical characteristics of water nanodroplets with various Weber numbers were studied at five translation velocities, four vibration amplitudes, and five vibration periods of the surface. The results show that when water nanodroplets impinge on translation surfaces, water molecules not only move along the surfaces but also rotate around the centroid of the water nanodroplet at the relative sliding stage. Water nanodroplets spread twice in the direction perpendicular to the relative sliding under a higher surface translation velocity. Additionally, a formula for water nanodroplets velocity in the translation direction was developed. Water nanodroplets with a larger Weber number experience a heavier friction force. For cases wherein water nanodroplets impinge on vibration surfaces, the increase in amplitudes impedes the spread of water nanodroplets, while the vibration periods promote it. Moreover, the short-period vibration makes water nanodroplets bounce off the surface.

## 1. Introduction

Droplets impinging on solid surfaces are pervasive in nature and industrial production, such as pesticide spraying [1], spray cooling, and inkjet printing [2,3]. A further understanding of this phenomenon is of great importance with the increasing demand for droplet manipulation, such as self-cleaning [4], anti-icing [5,6], and energy harvesting [7]. Although many efforts have been made in this field by researchers, the mechanisms of dynamical behaviors for impinging droplets are not fully clarified.

It is important to recognize that impinging droplets experience a process of spreading, retraction, and eventually bouncing off or depositing on surfaces [8,9,10]. Many experimental investigations have analyzed the dynamical behaviors of droplets. Wang et al. investigated water droplets impinging on pillar-arrayed polydimethylsiloxane (PDMS) surfaces with different solid fractions and suggested that the lower and upper limits of the Weber number (*We*) for the complete rebound of droplets decreased with solid fractions [11]. Hu et al. revealed the asymmetric spreading and retraction processes in the axial and spanwise directions when droplets impinged on ridged superhydrophobic surfaces. They proposed two theoretical models to quantitatively describe the spreading of droplets for size ratios (the ratio of the ridge diameter to the droplet diameter) as smaller and larger than the critical size ratio [12]. Work by Refael et al. showed the dynamical behaviors of droplets impinging on moving liquid surfaces. Droplets can splash, coalesce with, or bounce off liquid surfaces, which depends on the liquid properties and the velocity of droplets and surfaces [13]. Almohammadi et al. investigated the asymmetric spreading of droplets impinging on a moving surface and developed a model predicting the shape of the lamella. They also discussed the effect of surface wettability on droplets in the retraction process [14].

Recently, the exploration of nanodroplets has become a highly debated topic due to the development of micro and nano technology, such as nanoprinting and nanocoating. However, many important details cannot be displayed in experiments. Molecular dynamics (MD) simulations provide a powerful tool to probe the dynamical behaviors of nanodroplets on an atomic scale. The mechanisms of spreading, break-up, bouncing, and other behaviors of nanodroplets are discussed via MD [15,16,17,18,19]. Chen et al. simulated polymer nanodroplets impinging on a solid surface and found that the viscous dissipation of water nanodroplets stemmed from the velocity gradients in both the impinging and spreading directions [20]. Song et al. investigated the deformation behaviors of water nanodroplets in an electric field and observed the deformation hysteresis phenomenon. The distribution of average dipole orientations in water molecules also showed hysteresis [21]. Kwon et al. presented the dynamical behaviors of a water nanodroplet impinging on a stepped surface with different wetting gradients and step heights [22]. They found three phenomena: fully climbing the step, partially climbing the step, and being blocked by the step, which relied on the normalized step height. MD simulations were also adopted to investigate the influences of surfaces vibrating with high frequency on the wetting mechanism, evaporation, and transportation of nanodroplets [23,24,25,26].

However, studies on water nanodroplets impinging on moving solid surfaces are still scarce, especially on high-frequency vibrating surfaces. Due to the rapid development of piezoelectric materials, the frequency of surface acoustic waves (SAW), based on the principle of the inverse piezoelectric effect [27], is up to the level of GHz [28,29,30,31,32,33]. Therefore, high vibrations of surfaces with a frequency on the order of GHz can be realized. In this paper, the dynamical behaviors of water nanodroplets impinging on translation and vibration surfaces are investigated through MD simulations, respectively. The effects of parameters related to translation and vibration on the dynamical behaviors are explored.

## 2. Simulation Model and Methodology

**Figure 1:**
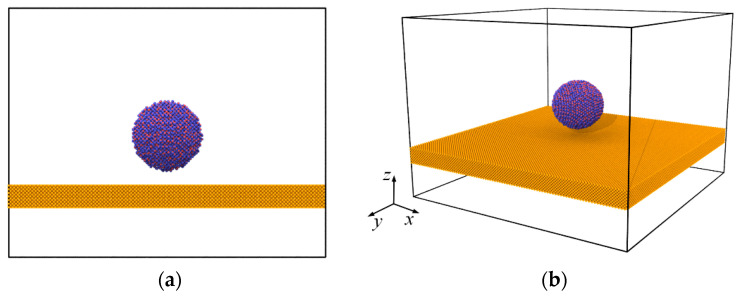
The initial configuration of a water nanodroplet impinging on a moving surface: (**a**) front view; (**b**) perspective view.

The initial configuration of water nanodroplets impinging on a moving surface is schematically shown in Figure 1. The flat substrate is composed of copper atoms, which are arranged in the face-centered-cube (FCC) structure with a lattice constant of 3.615 Å [34]. A TIP4P model of water is adopted because it can present more accurate dynamical characteristics [35,36]. The parameters corresponding to the TIP4P model are listed in Table 1 (*q*_O_: the charge of oxygen atom; *q*_H_: the charge of hydrogen atom; *e*: electron charge; *r*_OH_: the bond length between oxygen and hydrogen atoms; *θ*_HOH_: the bond angle of hydrogen-oxygen-hydrogen). The water nanodroplet is constituted by 5991 water molecules, with 35 Å in radius. To prevent atoms in the initial configuration from overlapping, which leads to the infinite interaction force among atoms, water molecules are arranged in the body-centered-cube (BCC) structure, whose lattice constant is determined by the density of water *ρ* at 298 K.

The whole simulation is programmed and conducted with LAMMPS (large-scale atomic/molecular massively parallel simulator) [37]. Periodical boundary conditions are applied in all three directions. A SHAKE algorithm is adopted to fix the bond length and angle of water molecules [38]. Copper atoms are constrained to initial equilibrium position during simulation [39,40,41]. The interaction between water molecules [42], containing a 12-6 Lennard-Jones (LJ) term and a long-range Coulomb term, is shown as follows:

(1) $$U=4\varepsilon_{\mathrm{OO}}\left[{\left(\frac{\sigma_{\mathrm{oo}}}{r_{\mathrm{oo}}}\right)}^{12}-{\left(\frac{\sigma_{\mathrm{oo}}}{r_{\mathrm{oo}}}\right)}^{6}\right]+\overset{3}{\underset{a=1}{\sum }}\overset{3}{\underset{b=1}{\sum }}\frac{q_{ia}q_{jb}}{4\pi \varepsilon_{0}r_{ia,jb}}$$

where *r*_OO_ denotes the distance between oxygen atoms in water molecules *i* and *j*. *ε*_OO_ and *σ*_OO_ represent the well depth and equilibrium distances, respectively. *r_ia,jb_* stands for the distance between charge site *a* of molecule *i* and charge site *b* of molecule *j*. *q_ia_* and *q_jb_* are charges of sites *a* and *b*, respectively. *ε*_0_ denotes the vacuum permittivity. Although Lennard-Jones potential was presented for noble gas at first, it essentially describes the interaction between electronic neutral atoms [43]. Hendrik et al. justified the reliability of this potential for copper and showed that 12-6 LJ potential is more suitable for large simulation systems (~10^6^ atoms) compared with embedded atom model (EAM) and density functional method [44]. In this paper, a large-sized solid surface consisting of 201,344 copper atoms is adopted to avoid the influences of adjacent mirror images during the spreading of the droplet. Therefore, both the interactions between copper atoms and those between oxygen atoms and copper atoms are implemented by using the 12-6 LJ potential [45,46,47]. The interaction parameters for oxygen and copper are presented in Table 2. Since the solid surface is frozen in the simulation, the parameters of copper atoms are mainly used to calculate the interaction between copper and oxygen through Lorentz–Berthelot mix rules that are shown in Equations (2) and (3).

(2) $$\sigma_{mn}=\frac{1}{2}\left(\sigma_{m}+\sigma_{n}\right)$$

(3) $$\varepsilon_{mn}=\sqrt{\varepsilon_{m}\varepsilon_{n}}$$

where *σ_m_*, *ε_m_*, *σ_n_*, and *ε_n_* are parameters of 12-6 LJ potential for atoms *m* and *n*; *σ_mn_* and *ε_mn_* are parameters of 12-6 LJ potential between atoms *m* and *n*.

The cutoff distances of 12-6 LJ potential and Coulomb potential are 10 Å and 12 Å [34], respectively. Velocity–Verlet algorithm is used to integrate the Newton equation of motion and update the velocities and coordinates of atoms with a time step of 1.0 fs. The long-range Columbic interaction is calculated by PPPM (particle–particle–particle–mesh) method in *K* space [49].

Water nanodroplets experience minimization and relaxation lasting for 0.5 ns in NVT ensemble, leading them equilibrated and the potential energy being the local minimum. During the relaxation process, a Nose–Hoover thermostat is adopted to realize temperature control and the initial equilibrium velocity of water molecules is in accord with Maxwell–Boltzmann distribution [50,51]. After relaxation, the simulation system achieves equilibrium state, and there are some water molecules in gas phase randomly distributed in simulation domain. Then, the NVT ensemble is removed, eliminating the temperature control, and the system is simulated under NVE ensemble. Meanwhile, a vertical initial velocity *V_z_* is applied to the water nanodroplet. *V_z_* is in the negative *z* direction. The moment water nanodroplets touch the solid surface is considered 0 ps.

## 3. Results and Discussion

### 3.1. Calculation of the Contact Angle and Verification of Water Nanodroplet Size

**Figure 2:**
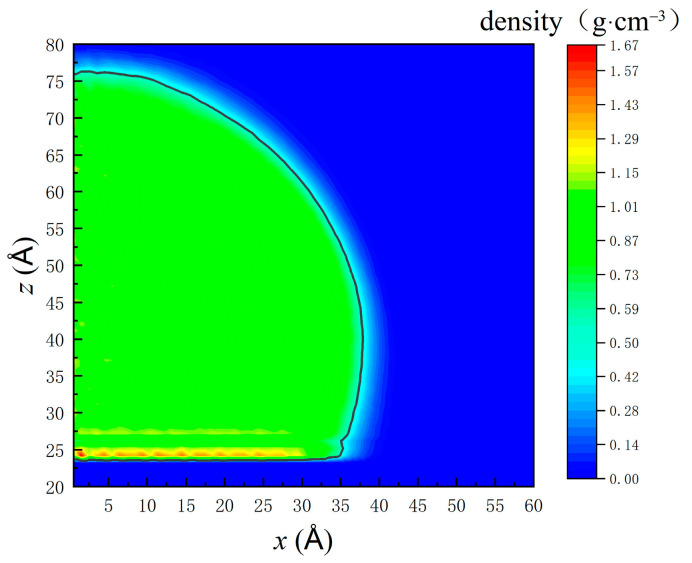
Density profile of the right-sided water nanodroplet on the smooth copper surface.

The contact angle is an essential parameter indicating the wettability of solid surfaces. The density contour of a water nanodroplet on the smooth copper surface illustrated in Figure 2 is based on the time-average density of every single chunk the water nanodroplet is being divided into. Considering the symmetrical projection of the water nanodroplet on *x–y* plane, only the right-sided water nanodroplet is investigated for contact angle calculation. The curve where the density is half that of bulk water is selected as the boundary of the liquid and gas phase (the black line in Figure 2) [52]. The density profile is considered as a part of a circle and is fitted by Equation (4). The contact angle can be obtained through Equation (5) [48]:

(4) $${\left(x-a\right)}^{2}+{\left(z-b\right)}^{2}=R^{2}$$

(5) $$\theta =arcsin\left(\frac{b-z_{\mathrm{sub}}}{R}\right)+90^{o}$$

where *a* and *b* are *x* and *z* coordinates of the centroid and *R* denotes the radius of the fitted circle. Only chunks that are more than 5 Å above the solid surface are considered to avoid the effects of density fluctuations at the liquid–solid interface. Therefore, *z*_sub_ is the *z* coordinate of a virtual surface 5 Å above the solid surface. According to the calculation, the equilibrium contact angle *θ* of water droplets on the smooth copper surface is 108.7°, and the corresponding experimental result is 102° [53]. The slight difference between them is attributed to the surface used in the experiment being not as smooth as that used in the simulation.

**Figure 3:**
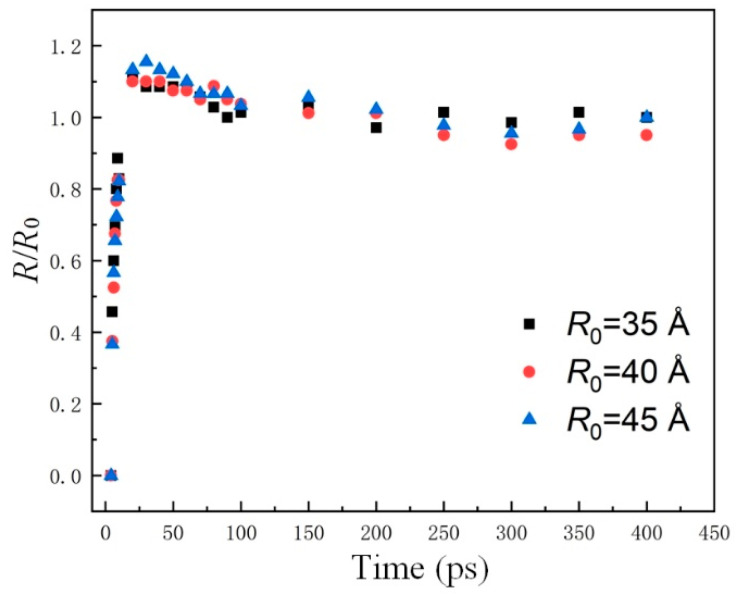
Effects of droplets size on spreading time.

To explore the effect of the number of molecules on the dynamical behaviors, nanodroplets with different radii impinging on the immobile surface are simulated. Radii and the corresponding number of water molecules are listed in Table 3. The dimensionless number (*R*/*R*_0_, *R* is the spreading radius, and *R*_0_ represents the initial radius) of those nanodroplets is shown in Figure 3.

It can be seen that although the number of water molecules varies, the *R*/*R*_0_ of different nanodroplets are similar to each other, and the maximum relative error is 6.9%. Therefore, the effect of the number of molecules arranged in the droplet can be neglected. Taking the computation accuracy and efficiency into consideration, nanodroplets with *R*_0_ = 35 Å are adopted.

### 3.2. Effects of Translation Velocity When Water Nanodroplets Impinge on Translation Surfaces

**Figure 4:**
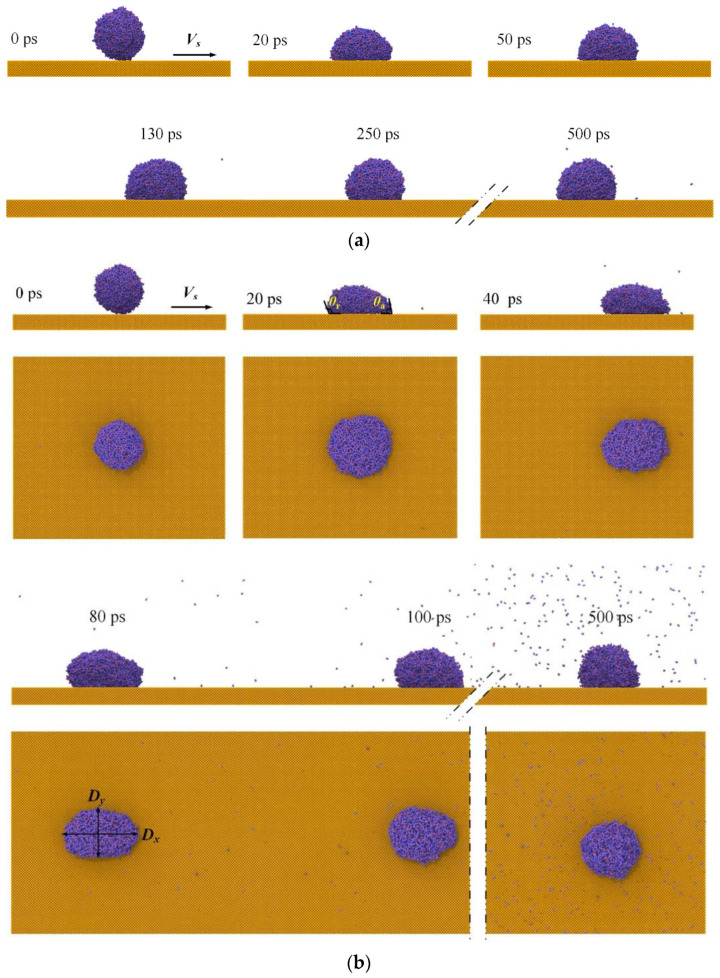
Morphological evolution of droplets impinging on surfaces with different translation velocities: (**a**) *V_s_* = 0.5 Å/ps; (**b**) *V_s_* = 9 Å/ps.

Considering the inertia force plays an important role in the impinging process, the Weber number (*We*) is chosen to represent the characteristics of water nanodroplets [54]. The effects of translation velocity of surfaces *V_s_* on the dynamical behaviors of water nanodroplets with *We* = 7.41 are investigated. *We* is defined as

(6) $$We=\frac{\rho RV_{z}^{2}}{\gamma }$$

where *R* is the radius of the droplet, *V_z_* represents the velocity of droplets, and the surface tension *γ =* 72.75 mN/m [52]. The morphological evolution of water nanodroplets is illustrated in Figure 4. Due to the sufficient simulation period, water nanodroplets can exceed the simulation box boundary, and they re-enter the box from the opposite side because of the periodical boundary conditions. For the sake of clarity, the dimensions of boxes and surfaces in *x* direction are assumed to be infinite.

Although the surface is smooth, Figure 4 shows that both deformation and displacement of water nanodroplets can be observed, which is due to microscopic forces such as the Van der Waals force and capillary force taking a leading role in the interaction on a nanoscale. The component of *F*, interaction force between the water nanodroplet and surface, in the direction of relative sliding, is defined as friction force *F_x_*, and the component in the direction perpendicular to the contact area is defined as normal pressure *F_z_*. Therefore, the deformation and displacement of water nanodroplets result from the *F_x_* applied by moving surfaces.

As seen in Figure 4a, the morphology of the water nanodroplet remains nearly symmetrical in the spreading and retraction processes. With *V_s_* increasing to 9 Å/ps, contact angle hysteresis is observed during the process, so that the water nanodroplet moves along the *V_s_* direction and spreads. Contact angle hysteresis refers to the difference between the advancing contact angle *θ_a_* and the receding contact angle *θ_r_*. The extent of hysteresis experiences a gradual drop to zero as time passes, which means the contact angle hysteresis eventually disappears. The water nanodroplet spreads asymmetrically in *x* and *y* directions at time *t* = 40 ps, and the asymmetry becomes more significant as time elapses. In particular, the water nanodroplet evolves into an ellipsoid at *t* = 80 ps. Afterwards, the water nanodroplet gradually recovers the sphere shape under the action of surface tension and then moves alongside the surface.

**Figure 5:**
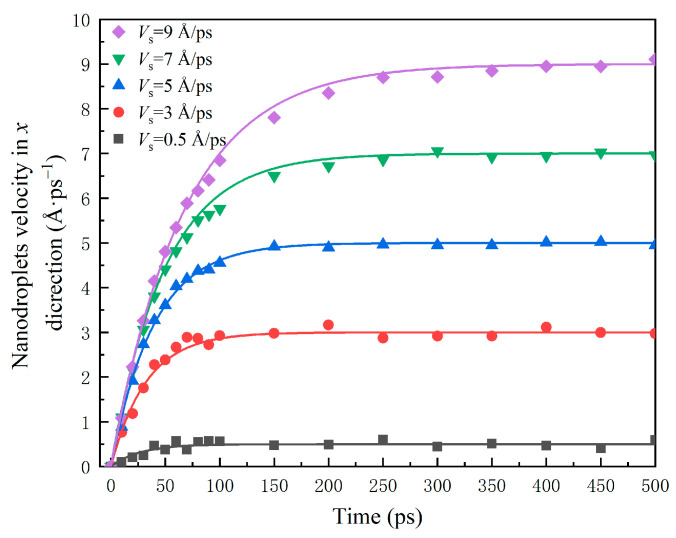
Time evolution of velocity of the water nanodroplet in the *x* direction.

The evolution of velocity of the water nanodroplet in the *x* direction, *V_x_*, is shown in Figure 5. It can be seen that the variation of *V_x_* can be divided into two stages: the relative sliding stage and the stable stage. At the relative sliding stage, the acceleration of *V_x_* increases with the surface velocity. Especially, during 0~50 ps, *V_x_* almost linearly increases with *t*. This is because the deformation of water nanodroplets becomes more severe with the increase in surface velocity, making the spreading area enlarged, and thus the interaction between droplets and surfaces is enhanced. Once the water nanodroplet reaches the maximum spreading state, it begins to retract, and the interaction becomes weakened, resulting in a slowdown in the acceleration of *V_x_*. At a stable stage, the translation velocity of the water nanodroplet is roughly equal to that of the surface, and there is no relative sliding between them.

The mathematical expression, presented in Equation (7), quantitatively describes the change in *V_x_* in the whole dynamical process. Water nanodroplets with *We* ranging from 7.41 to 66.67 impinging on the translation surface are also simulated, and this equation can provide good approximations.

(7) $$V_{x}=V_{s}-V_{s}{\left(0.962+0.019\frac{V_{s}}{We}\right)}^{t}\;\;\;7.41\leq We\leq 66.67$$

**Figure 6:**
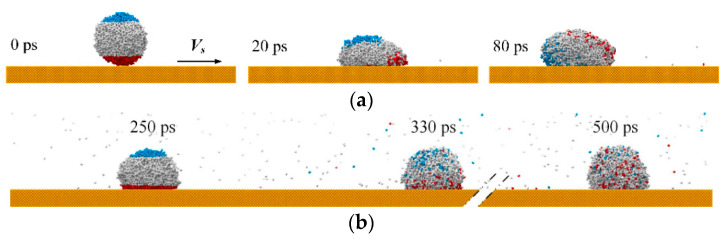
Different motion mechanisms of water molecules inside the water nanodroplet at two stages: (**a**) relative sliding stage; (**b**) stable stage.

Taking the surface with *V_s_* = 9 Å/ps as an example, the motion mechanisms of water molecules at different stages are explored. According to Figure 5, the water nanodroplet is at a relative sliding stage during 0~250 ps and at a stable stage during 250~500 ps. As illustrated in Figure 6a, the top and bottom sections of the water nanodroplet are colored blue and red at *t* = 0 ps, respectively. The color property is fixed during 0~250 ps. According to the snap shots, water molecules in the blue and red regions rotate counterclockwise around the center of the water nanodroplet, accompanying the diffusion of water molecules into the entire water nanodroplet. Then, the water nanodroplet is colored again at 250 ps, as shown in Figure 6b. During the period of 250~500 ps, water molecules in blue and red regions only move downward or upward due to the diffusion, and no rotation is observed. Therefore, diffusion aside, water molecules in the water nanodroplets rotate around the centroid while they move along the surface in the relative sliding stage, and the rotation of water molecules disappears in the stable stage.

**Figure 7:**
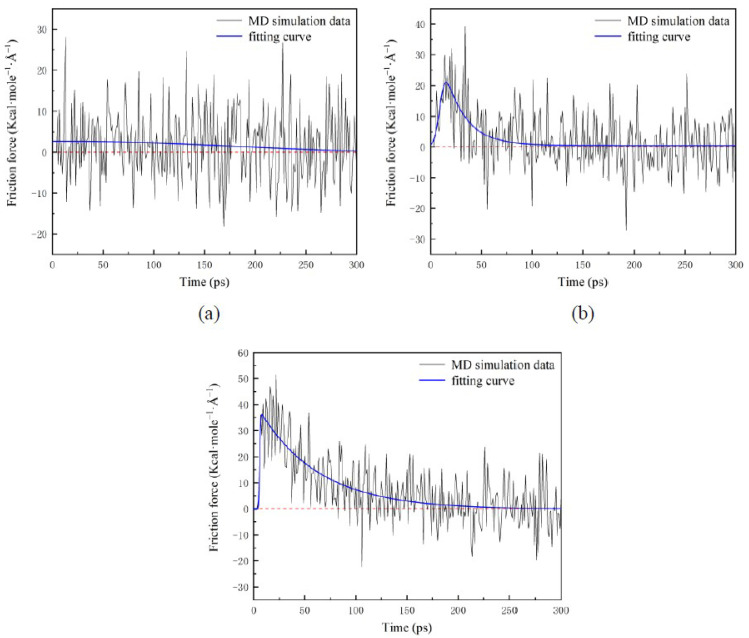
Time evolution of the friction force applied to droplets: (**a**) *V_s_* = 0.5 Å/ps; (**b**) *V_s_* = 3 Å/ps; (**c**) *V_s_* = 9 Å/ps.

Friction force *F_x_* applied to water nanodroplets is shown in Figure 7. When the surfaces translate at high speed (e.g., *V_s_* = 9 Å/ps), there are continuous positive values of *F_x_* in a long period, which is about 80 ps, as shown in Figure 7c. Additionally, that period shrinks as *V_s_* decreases, as illustrated in Figure 7a,b. As *V_s_* increases to 3 and 9 Å/ps from 0.5 Å/ps, the maximum friction force climbs to 20.5 and 36 Kcal·mole^−1^·Å^−1^, which means that *F_x_* increases with the *V_s_*. In the stable stage, *F_x_* fluctuates around 0. The fluctuation results from the random thermal motion that leads to a slight change in the spreading area.

**Figure 8:**
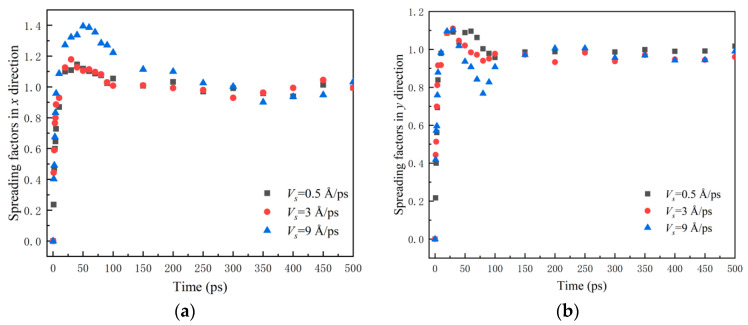
Time evolution of spreading factors in *x* and *y* directions: (**a**) *β_x_*; (**b**) *β_y_*.

Figure 8 shows the spreading factors *β_x_* and *β_y_*. *β_x_* (*β_y_*) denotes the ratio spreading length *D_x_* (*D_y_*) of water nanodroplets in the *x*(*y*) direction to the initial diameter *D*_0_. *β_x_*_max_ (*β_y_*_max_) is the maximum of *β_x_* (*β_y_*).

For the case *V_s_* = 9 Å/ps, it can be observed from Figure 8b that *β_y_* reaches its maximum at 30 ps and then descends to 0.76 rapidly at 80 ps. After, it climbs again and finally obtains stability. This inflection point indicates the water nanodroplet spreads twice in the *y* direction, which is perpendicular to the direction of relative sliding. This is due to the fact that the water nanodroplet suffers reactive force when it impinges on solid surfaces, and that makes the water nanodroplet spread at first. The *β_y_*_max_ is realized soon, and then the water nanodroplet begins to retract in the *y* direction. Meanwhile, the tensile deformation of water nanodroplets in the *x* direction becomes severe with the increase in *V_x_* and thus accelerates the retraction in the *y* direction since the volume of the water nanodroplet is constant. Once the *β_x_*_max_ is realized, the water nanodroplet begins to retract in the *x* direction, which results in the water nanodroplet spreading again in the *y* direction.

### 3.3. Effects of the Weber Number When Water Nanodroplets Impinge on Translation Surfaces

To investigate the influence of *We* on the behavioral evolution, the water nanodroplet with *We* ranging from 0.82 to 66.67 hitting the translation surfaces is simulated. The solid surface translates at the speed of *V_s_* = 7 Å/ps along the positive direction of *x* axis.

**Figure 9:**
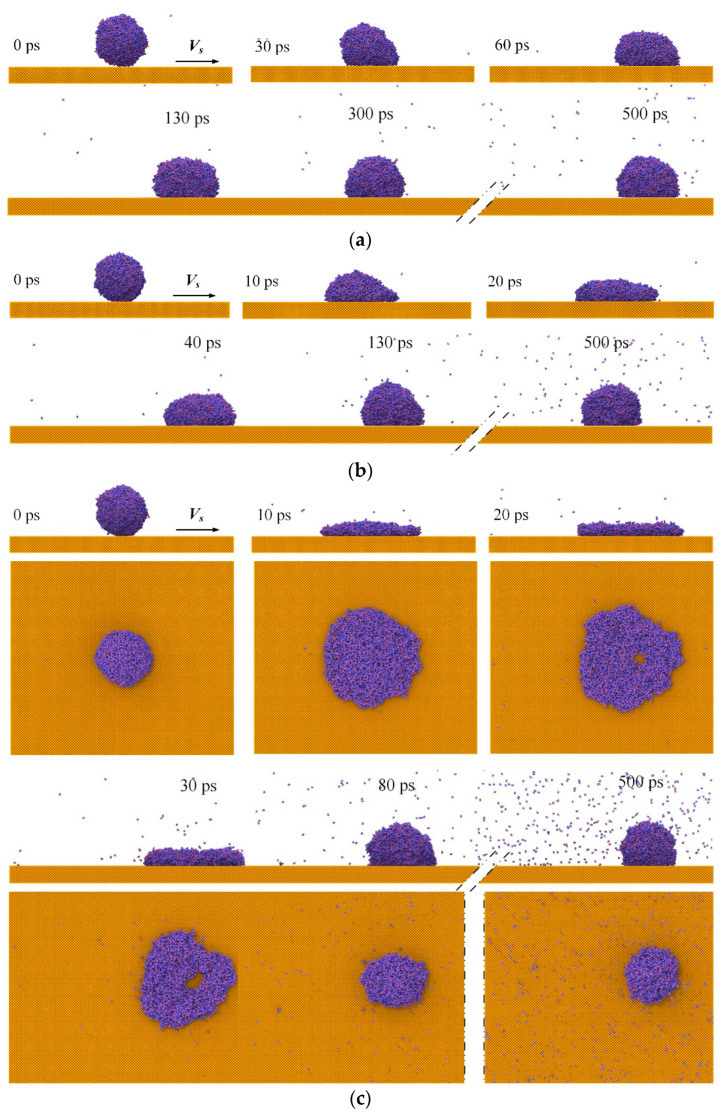
Morphological evolution of water nanodroplets with various *We* impinging on the translation surfaces: (**a**) *We* = 0.82; (**b**) *We* = 20.58; (**c**) *We* = 66.67.

The performance of water nanodroplets is shown in Figure 9. It can be observed that water nanodroplets with different *We* lie on various positions of the surfaces despite the identical *V_s_*, which suggests the dynamical behaviors of water nanodroplets are determined by the joint actions of *V_s_* and *We*.

The contact hysteresis can be seen for water nanodroplets with *We* = 0.82~20.58, as shown in Figure 9a,b. However, the degree of hysteresis decreases with increasing *We,* and the hysteresis cannot be observed for *We* = 66.67, as shown in Figure 9c. This can be explained by the fact that when *We* is relatively low, the spreading velocity after impinging is also low due to inertia, which makes the influence of friction force more remarkable, and thus the tensile deformation becomes more obvious. Therefore, the contact angle hysteresis is more remarkable. As *We* increases, the spreading velocity after impinging increases, and the friction force plays a less important role in the dynamical behaviors. As a consequence, the difference between *θ_a_* and *θ_r_* decreases gradually. It is important to note that a hole appears near the center of the water nanodroplet during 20~30 ps, as shown in Figure 9c, because the higher *We* facilitates the spreading of water nanodroplets, and the water molecules move towards the edge of the water nanodroplet constantly during the spreading process. However, the hole disappears under the action of surface tension during the retraction process.

**Figure 10:**
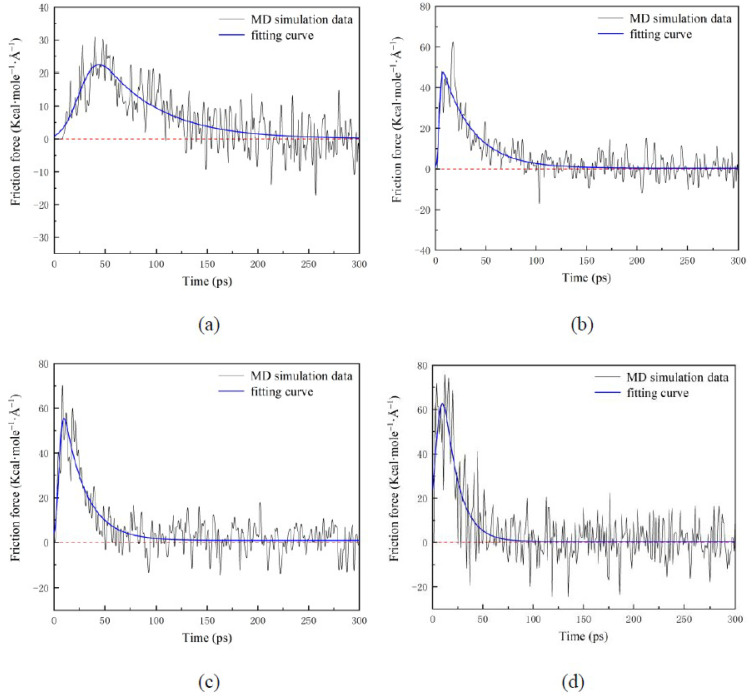
Time evolution of the friction force applied to water nanodroplets with various *We*: (**a**) *We* = 0.82; (**b**) *We* = 20.58; (**c**) *We* = 40.33; (**d**) *We* = 66.67.

Figure 10 illustrates the friction force *F_x_* applied to water nanodroplets with various *We*. *F_x_* increases with *We* at the relative sliding stage, and thus, the water nanodroplet comes into the stable stage early, where the water nanodroplet and surface have the same velocity. This is because the increased spreading area leads to the enhancement of the interaction between water and solid surfaces.

**Figure 11:**
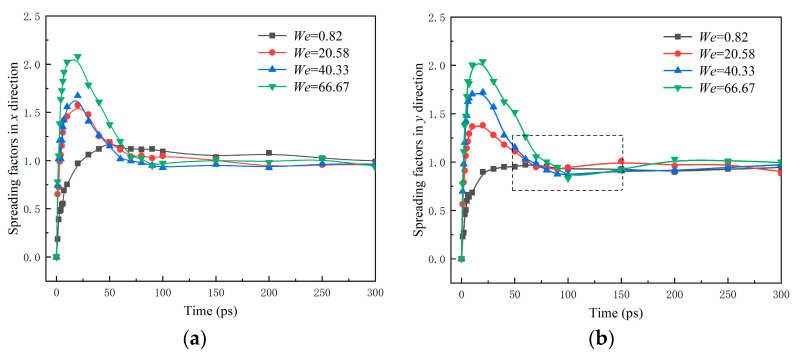
Time evolution of spreading factors in *x* and *y* directions: (**a**) *β_x_*; (**b**) *β_y_*.

From the spreading factors shown in Figure 11, it can be seen that both *β_x_* and *β_y_* are approximately 1.0 after 150 ps despite the different *We*, which indicates that *We* has little influence on the steady state of water nanodroplets and mainly takes a role in the spreading and retraction processes. In addition, *β_x_*_max_ and *β_y_*_max_ increase with the *We*. It should be noted that in the dashed line region shown in Figure 11b, besides *We* = 0.82, *β_y_* decreases first and then gradually increases to a stable level. Therefore, the water nanodroplet experiences a secondary spreading in the *y* direction.

### 3.4. Effects of Vibration Amplitudes on Dynamical Behaviors When Water Nanodroplets Impinge on Vibration Surfaces

**Figure 12:**
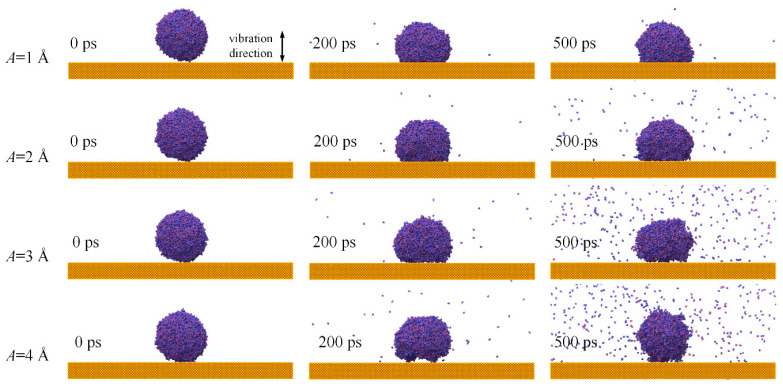
Morphological evolution of water nanodroplets impinging on vibration surfaces with various amplitudes.

To explore the influence of vibration amplitudes *A* on the dynamical behaviors, water nanodroplets impinging on surfaces with vibration amplitudes ranging from 1 to 4 Å, and the vibration period *T* = 12 ps is simulated. Vibration is achieved by applying periodic displacement up and down along the *z* axis on the solid surface, as shown in Figure 12.

The spreading area reduces with the increasing *A*, as shown in Figure 12. In particular, when *A* = 3 and 4 Å, there are some gaps between the water nanodroplets and surfaces. Hence, the adhesion between the water nanodroplet and surface is weakened, and more water molecules escape from the bulk of water nanodroplets with the increase in *A*.

**Figure 13:**
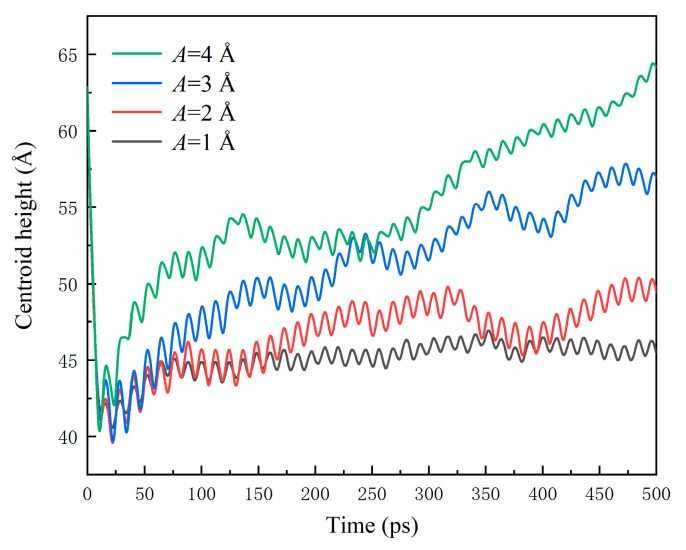
Centroid height of water nanodroplets impinging on vibration surfaces with various amplitudes.

Figure 13 shows the centroid height *h* of water nanodroplets. In general, *h* increases, and water nanodroplets have a tendency to depart from surfaces as *A* increases. During 0~20 ps, *h* decreases rapidly. That is because in this period, inertia is the dominant factor determining the dynamical behaviors, and the water nanodroplet spreads at high speed, and as a result, *h* almost decreases linearly. Once *h* decreases to the minimum, indicating the maximum spreading state being realized, the water nanodroplet starts to retract due to surface tension. However, energy dissipation in the spreading process leads to a low retraction velocity. Hence, the vibration of surfaces gradually comes into play, and the saw-tooth fluctuation appears in a single vibration period. After 70 ps, the retraction almost finishes, and the dynamical behaviors of water nanodroplets are solely affected by the vibration surfaces. The amplitudes of saw-tooth fluctuations decrease, and the shape of the saw tooth becomes uniform.

**Figure 14:**
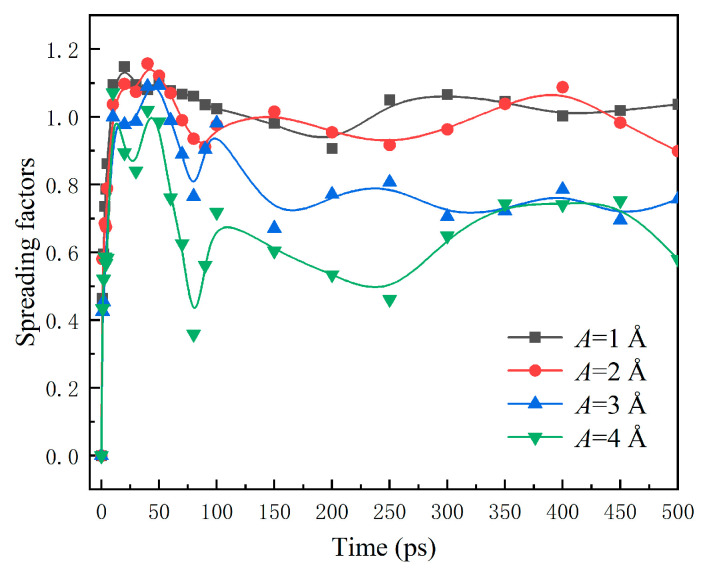
Time evolution of spreading factors of droplets impinging on surfaces with different vibration amplitudes.

The variation of spreading factors *β* with time is recorded in Figure 14. It also shows apparent fluctuation due to the vibration surfaces. The maximum spreading factor *β*_max_ and the maximum spreading area reduce with the increasing *A*, suggesting a larger vibration amplitude not conducive to the spreading of water nanodroplets.

### 3.5. Effects of Vibration Periods on Dynamical Behaviors When Water Nanodroplets Impinge on Vibration Surfaces

A water nanodroplet with *We* = 7.41 impinging on vibration surfaces with periods *T* ranging from 2 to 16 ps and *A* = 2 Å is simulated to analyze the influence of *T* on dynamical behaviors.

**Figure 15:**
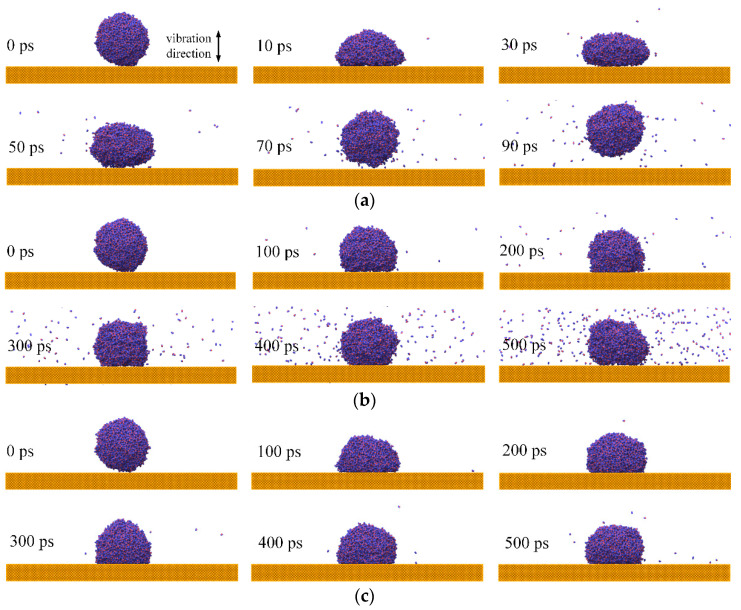
Dynamical behaviors of water nanodroplets impinging on surfaces with different vibration periods: (**a**) *T* = 2 ps; (**b**) *T* = 8 ps; (**c**) *T* = 16 ps.

Figure 15 presents the snapshots in that process. Water nanodroplets eventually deposit on the solid surfaces when *T* = 4~16 ps. It can be seen that a longer *T* makes the connection between water nanodroplets and solid surfaces tighter, e.g., *T* = 16 ps in Figure 15c. It should be noted that the water nanodroplet finally bounces off the solid surface when *T* = 2 ps. This provides a method to facilitate water nanodroplets bouncing, except when designing special rough structures on the surfaces mentioned in the previous study [55]. Moreover, the water nanodroplet has a shorter contact time with the solid surface than the alternative.

**Figure 16:**
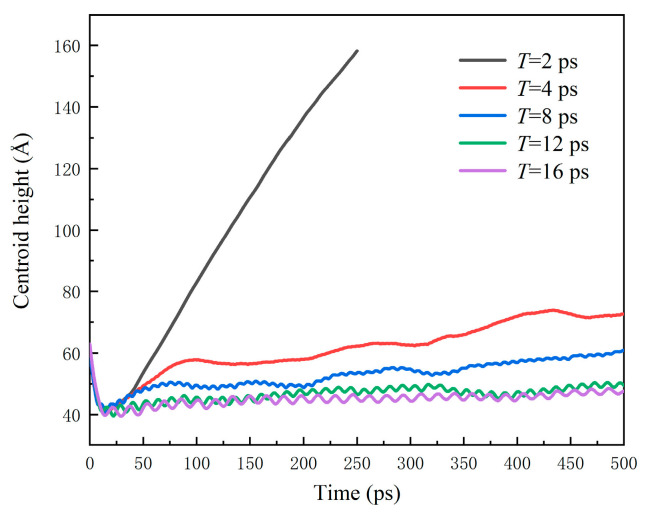
Time evolution of water nanodroplets impinging on surfaces with different vibration periods.

The variation in centroid height *h* for water nanodroplets is shown in Figure 16. *h* nearly remains stable after 25 ps, when *T* = 12 and 16 ps. As *T* decreases, *h* increases. Especially, the water nanodroplet bounces off the solid surfaces at 55 ps when *T* = 2 ps.

**Figure 17:**
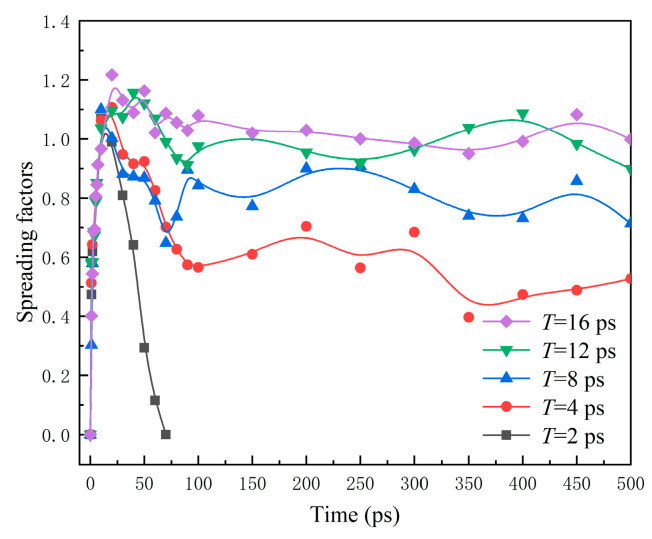
Spreading factors of water nanodroplets impinging on solid surfaces with various vibration periods.

According to Figure 17, as a whole, *β* decreases with the decrese in the vibration period, and even it finally decreases to 0 for *T* = 2 ps. As a result, the increasing *T* induces the further spread of the water nanodroplet.

**Figure 18:**
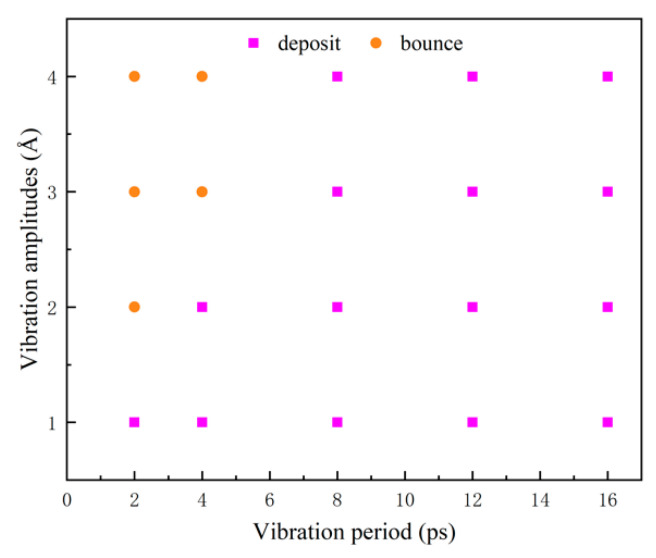
The bounce domain as water nanodroplets impinge on vibration surfaces.

To quantify the bounce of water nanodroplets, the bounce domain, determined by *T* and *A*, of water nanodroplets impinging on vibration surfaces, is showcased in Figure 18. This provides the theoretical basis for practical applications.

## 4. Conclusions

In this work, molecular dynamics simulation was used to investigate the dynamical behaviors of water nanodroplets impinging on the translation surfaces and vibration surfaces. The influences of the translation velocity of the surface, the *We* of water nanodroplets, the vibration amplitudes, and the vibration periods on the dynamical behaviors were investigated. The main conclusions are as follows:

(1) The expression of velocity for water nanodroplets in the direction of translation was developed, which quantitively describes the velocity evolution of water nanodroplets as they impinge on translation surfaces.

(2) At the relative sliding stage, water molecules rotate around the centroid of the water nanodroplet and move along the translation direction. At the stable stage, the rotation of water molecules disappears.

(3) The higher the translation velocity is, the larger the friction force applied to the water nanodroplets is, and thus, the asymmetric spreading is more apparent. A higher translation velocity results in the water nanodroplet spreading twice in the direction perpendicular to the relative sliding.

(4) The increase in vibration amplitudes impedes the spreading of water nanodroplets, while the increase in vibration periods facilitates it. Additionally, the bounce domain of water nanodroplets was mapped.

## Figures and Tables

**Table 1:** Parameters of TIP4P model [35].

*q*_O_ (*e*)	*q*_H_ (*e*)	*r*_OH_ (Å)	*θ*_HOH_ (°)
−1.0484	+0.5242	0.9572	104.52

**Table 2:** Interaction parameters for oxygen and copper atoms [48].

Atom Type	O	Cu
*ε* (kcal/mol)	*ε*_O_ = 0.1628	*ε*_Cu_ = 0.2379
*σ* (Å)	*σ*_O_ = 3.1644	*σ*_Cu_ = 2.3400

**Table 3:** Nanodroplets with different radii and the number of water molecules.

Nanodroplet Radius (Å)	Number of Molecules
35	5991
40	8953
45	12,721

## Data Availability

The data related to this investigation are available on reasonable request.
